# The influence of another’s actions and presence on perspective taking

**DOI:** 10.1038/s41598-024-55200-8

**Published:** 2024-02-29

**Authors:** Ieva Lukošiūnaitė, Ágnes M. Kovács, Natalie Sebanz

**Affiliations:** 1 Department of Cognitive Science, Central European University, Vienna, Austria; 2 School of Psychology, University of East Anglia, Norwich, United Kingdom

**Keywords:** Psychology, Human behaviour

## Abstract

The ability to take each other’s visuospatial perspective has been linked to people’s capacity to perceive another’s action possibilities and to predict their actions. Research has also shown that visuospatial perspective taking is supported by one’s own mental own-body transformation. However, how these two processes of action perception and visuospatial perspective taking might interact remains largely unknown. By introducing seven angular disparities between participants and the model in the stimuli pictures across “Action” and “No Action” conditions, we investigated whether the observation of a goal-directed action facilitates perspective taking and whether this facilitation depends on the level of mental own-body transformation required to take perspective. The results showed that action observation facilitated performance independently of the level of mental-own body transformation. The processes behind this facilitation could involve anatomical mapping that is independent of the congruency between the participants’ and the model’s perspectives. Further, we replicated previous research findings, showing that participants were more accurate and faster when taking the perspective of a person compared to an inanimate object (a chair). The strongest facilitation effects were seen at the highest angular disparities between participants and the model in the stimuli pictures. Together, these findings enhance our knowledge of the mechanisms behind visuospatial perspective taking.

## Introduction

Being able to take each other’s visuospatial perspective is important for many social interactions^1^. Even when we share the same environment with others who are co-present, are surrounded by the same objects, and see the same things as they do, the exact perspective from which we perceive our surroundings and how we are situated relative to objects in our surroundings often differs from that of others. In order to successfully interact with each other, people need to readily estimate or imagine how one or another object looks to another person, where it is placed in relation to them, and whether and how they are able to act on it. This involves the capacity to mentally place oneself into another’s position in space to generate mental representations of a visual scene from their spatial location^2^.

A substantial amount of research has investigated the processes that underlie visuospatial perspective taking and the factors that influence it. First, it has been consistently shown that the dominant strategy to take another’s spatial perspective is mentally transforming and rotating ourselves into their position^3–5^. This strategy is reflected by increased reaction times with an increased angular disparity between participants and the person whose perspective they are taking^4–7^. Reaction times increase the most after 60–90 degrees of angular disparity between participants and another person whose perspective they are trying to take. At lower angular disparities, people can determine how objects are situated with respect to another person through visual matching since their perspective is largely congruent with the other's^4^.

Other research has connected perspective taking to the ability to perceive another’s action possibilities and to predict their actions^8^. The results of previous studies support this link and have provided evidence that action observation makes us more prone and faster to take another’s perspective. For example, introducing depictions of actions in stimuli pictures resulted in a higher proportion of responses that reflected considering another’s perspective as opposed to responses based on participants’ egocentric perspective^9–13^. Similarly, neurotypical participants were also faster to take another’s perspective when that person was grasping an object compared to when the person was sitting still, looking straight, or gazing directly at the object^9^. Knowing that another person is actively involved in a task also facilitated spontaneous visuospatial perspective taking in a stimulus–response compatibility task^14^. In addition, drawing attention to actions through specific questions resulted in a higher proportion of participants taking another’s perspective as opposed to their own^15^.

However, little is known about the specific mechanisms whereby action observation might facilitate perspective taking. The above-mentioned studies that investigated the influence of action observation on perspective taking were mainly based on measuring the proportion of spontaneous answers made by participants from another’s perspective and focused on 180° angular disparity between participants and the target person^9–13^. To achieve a better understanding of the processes whereby action observation facilitates perspective taking, in the present study, we asked participants to take the perspective of a model seated at seven different angular disparities. We measured reaction times and accuracy rates at these angular disparities in “Action” and “No Action” conditions, where the model in the stimulus pictures was either seen reaching for an object or sitting still. In this way, we aimed to investigate how action observation influences perspective taking at different levels of mental own-body rotation.

If the predicted facilitatory effects of observing another person acting are of the same magnitude for all the angular disparities between participants and the model in the stimuli pictures, this would suggest that the effects of action observation are independent of mental own-body rotation. In this case, we could conclude that seeing others acting facilitates judgments about the position of objects relative to their body (perhaps simply by priming a tendency to respond). At the same time, it would not affect the ease with which mental own-body rotation is performed.

On the other hand, an interaction between the “Action” and “No Action” conditions and angular disparity would indicate that action observation and mental own-body rotation depend on each other. If action observation facilitates mental own-body rotation or if facilitatory effects of action observation on perspective taking only occur when there is mental own-body rotation, we should see stronger effects for angles that require more mental own-body rotation (90°, 135°, 180°, 225°, and 270°) and weaker effects for the lowest angular disparities (45° and 315°).

In addition, we also aimed to replicate previous research findings (Kessler and Thompson^4^, Experiment 1 vs. 2) showing the importance of the presence of another person and the possible role of bodily mapping—covert imitation of another’s posture by mapping own body axes onto theirs^16,17^, in perspective taking. To this purpose, we introduced a “Person Absent” condition, in which no person was present, and instead, there was an empty chair in the scene. We expected slower reaction times in the “Person Absent” condition compared to a condition where a human figure was present. Based on findings by Kessler and Thompson (Experiment 1 vs. 2)^4^, an interaction with angular disparity might also be expected. They found that the differences in RTs between taking the perspective of a person and taking the perspective of an inanimate object were larger with increased angular disparities. This pattern could indicate that having a possibility for bodily mapping is more useful in perspective taking when more mental own-body rotation is needed. Specifically, having a human figure as a reference and mapping our bodily axes on them could help reduce the cognitive load required to mentally transform ourselves and sustain the image at the highest angular disparities.

Altogether, the present experiments aimed to answer the following questions: How does observing another’s actions affect perspective taking? Does action observation affect perspective taking in the same manner during different levels of mental own-body rotation? How does the possibility for bodily mapping affect perspective taking? To answer these questions, we ran three experiments. In Experiment 1a, participants judged the position of objects relative to a person sitting at a table who was either reaching for one of the objects (“Action” condition) or resting their hands on the lap (“No Action” condition). Experiment 1b included a no-person (“Person Absent”) condition. It investigated the importance of bodily mapping in perspective taking and was aimed at replicating previous findings by Kessler and Thompson (Experiment 1 vs. 2)^4^. In this experiment, we asked participants to take the perspective of an empty chair and compared it to the condition from Experiment 1a where the model was sitting on a chair with both of their hands on their lap. Experiment 2 aimed to replicate the results of the “Action” and “No Action” conditions in Experiment 1a with better-matched visual similarity between the conditions.

## Experiment 1a

**Figure 1:**
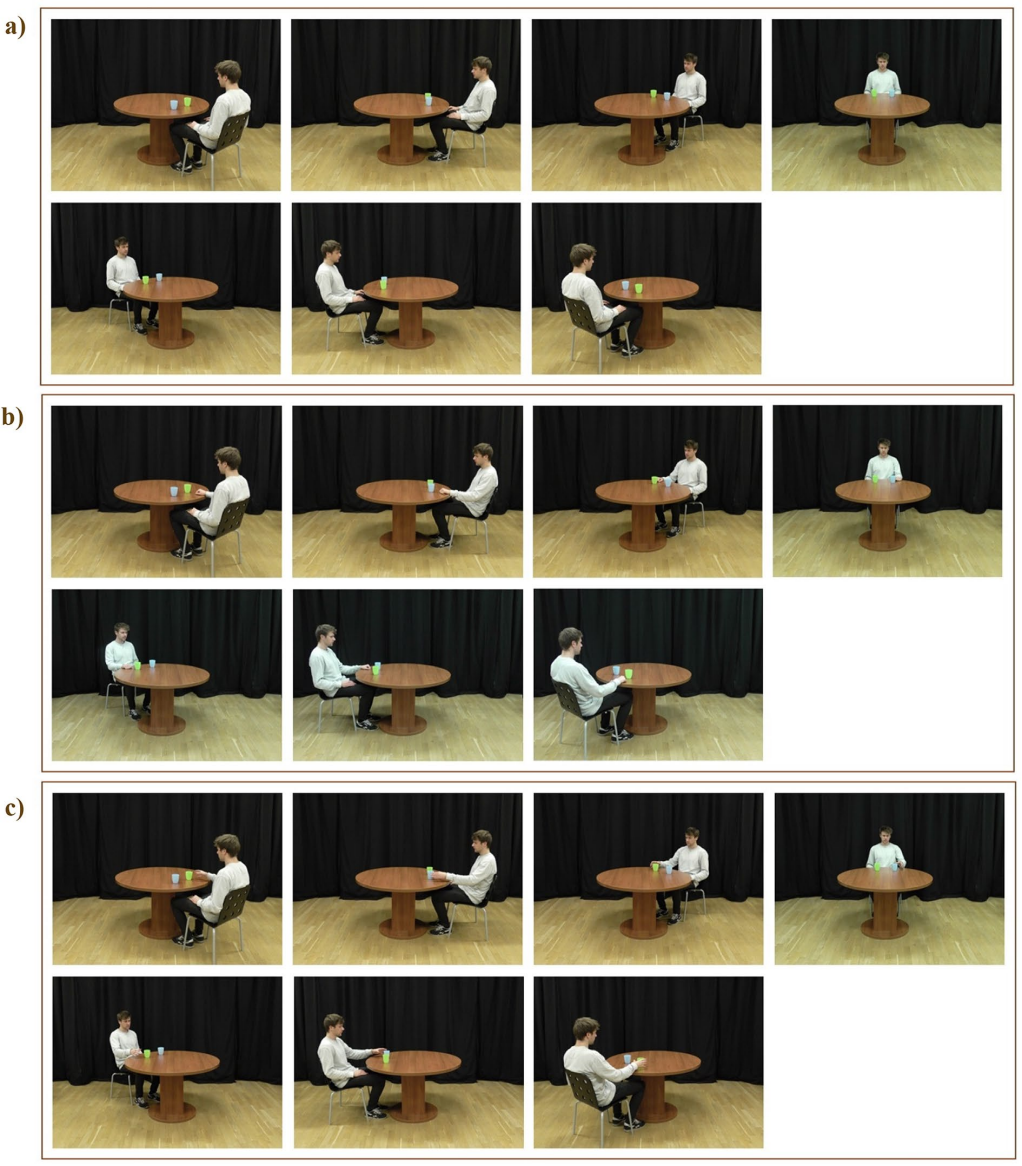
(**a**) Stimuli pictures in the “No Action” condition in Experiment 1a at all angles (from left to right, upper row: 45°, 90°, 135°, 180°; lower row: 225°, 270°, 315°); (**b**) Examples of the stimuli pictures in the “No Action” condition in Experiment 2; (**c**) Examples of the stimuli pictures in the “Action” condition in Experiments 1a and 2.

In Experiment 1a, we showed participants pictures of a person sitting at a round table at 7 different angles (45°, 90°, 135°, 180°, 225°, 270°, and 315°). There were two conditions. In the “Action” condition, the model in the pictures was reaching toward one of two objects (cups) (see Fig. 1c). The “No Action” condition resembled the “Action” condition, except that the model in the pictures was not reaching for a cup but had both hands on their lap (see Fig. 1a). Participants were asked to indicate whether the blue or green cup was to the left/right of the model. Whether participants saw the model using their right hand to reach for the cup on the right side or their left hand to reach for the cup on their left side was counterbalanced between participants; we did not predict any effects of hand used by the model. We expected that participants would be faster and more accurate in the “Action” condition compared to the “No Action” condition. We also predicted that participants would be slower and less accurate at higher angular disparities between them and the model in both conditions.

### Methods

#### Participants

Using an online platform for participant recruitment (Prolific, www.prolific.co), we recruited 80 participants. 40 of them (mean age = 21.67, *SD* = 5.31) were randomly assigned to the version of the experiment where the model was using the right hand (Group 1), and another 40 (mean age = 22.28, *SD* = 3.84) to the version of the experiment where the model was using the left hand (Group 2). Participants had normal or corrected-to-normal vision, were right-handed, and were between 18 and 35 years old.

To calculate sample size, we carried out a power analysis with the software program G*Power^18^ for a within-participant ANOVA, aiming at 0.8 power at the standard 0.05 alpha error probability with effect sizes of 0.20 and 0.18 for Group 1 and Group 2, respectively (Note: since our main prediction concerned the difference between the “Action” and “No Action” conditions, the G*Power analysis was carried out to identify the number of participants needed to detect a difference between these conditions, and did not include possible RT differences between different angular disparities or possible interaction effects between Angle and Condition). We estimated different effect sizes for Group 1 and Group 2 because of possible response interference effects in Group 2. Specifically, all participants were asked to press the response keys with their right hand. However, in Group 1, participants saw a right-hand action in the stimuli pictures in the “Action” condition, while in Group 2, a left-hand action was shown in the “Action” condition. This could have caused interference in the response times^19,20^. Therefore, we expected a slightly smaller effect size in Group 2 compared to Group 1. The power analysis showed that the number of participants required to achieve the specified power was 35 and 39 for Groups 1 and 2, respectively. To account for potential dropouts and outliers, we collected data from 40 participants in each group.

After data collection, participants with an accuracy rate of two standard deviations below the mean accuracy rate of all participants in each group were excluded from the data analyses. This led to two participants being excluded in Group 2. Therefore, in total, we analyzed the data of 78 participants.

The study was approved by the local Ethics committee (Psychological Research Ethics Board PREBO) and was conducted in accordance with the Helsinki Declaration^21^. Before the experiment, each participant was provided with information about the study and was asked to give informed consent. Participants were paid 6 GBP for their participation. Informed consent was obtained for the publication of identifying images in an online open-access publication.

#### Design

The experiment had a within-participant design with the factors: Condition (“Action” and “No Action”) and Angle (seven different angles: 45°, 90°, 135°, 180°, 225°, 270°, 315°).

#### Procedure and stimuli

The experiment was run online using the platform Prolific (www.prolific.co). The experiment was programmed using Psychopy software^22^. The data was collected through the platform Pavlovia (pavlovia.org).

First, the participants were asked to find a calm place to carry out the task and to use their personal laptops or stationary computers. Mobile phones or tablets were not allowed. After providing informed consent, the participants were presented with task instructions and were shown photograph stimuli depicting a male model sitting at a round table at seven different angles.

Participants in Group 1 were asked to respond “yes” or “no” to the question if one of the two objects is on the right of the person shown in the pictures. For example, “In the following trials, is the blue cup placed on the right of the person?”. Participants in Group 2 were asked to respond “yes” or “no” to the question if one of the two objects is on the left of the person shown in the pictures. For example, “In the following trials, is the blue cup placed on the left of the person?”. Participants used the keys “B” and “H” on the keyboard. Participants were randomly assigned to one of two key layouts: in Layout 1, the “B” key was assigned to the “yes” answer, and the “H” key was assigned to the “no” answer; in Layout 2, the “B” key was assigned to the “no” answer, while the “H” key was assigned to the “yes” answer.

Each participant completed 28 practice trials to get familiar with the task and to learn the response key assignment. During the practice trials, written feedback on the screen was provided. Participants saw the message ‘Correct!’ when they answered correctly, ‘Oops! That was wrong’ when the answer was incorrect, and ‘Oops! Missed’ when participants missed a trial. After the practice trials, participants proceeded to the task. They were informed that there would be no written feedback on the screen anymore and that instead, they would hear a short sound if they answered incorrectly or missed a trial.

**Figure 2:**
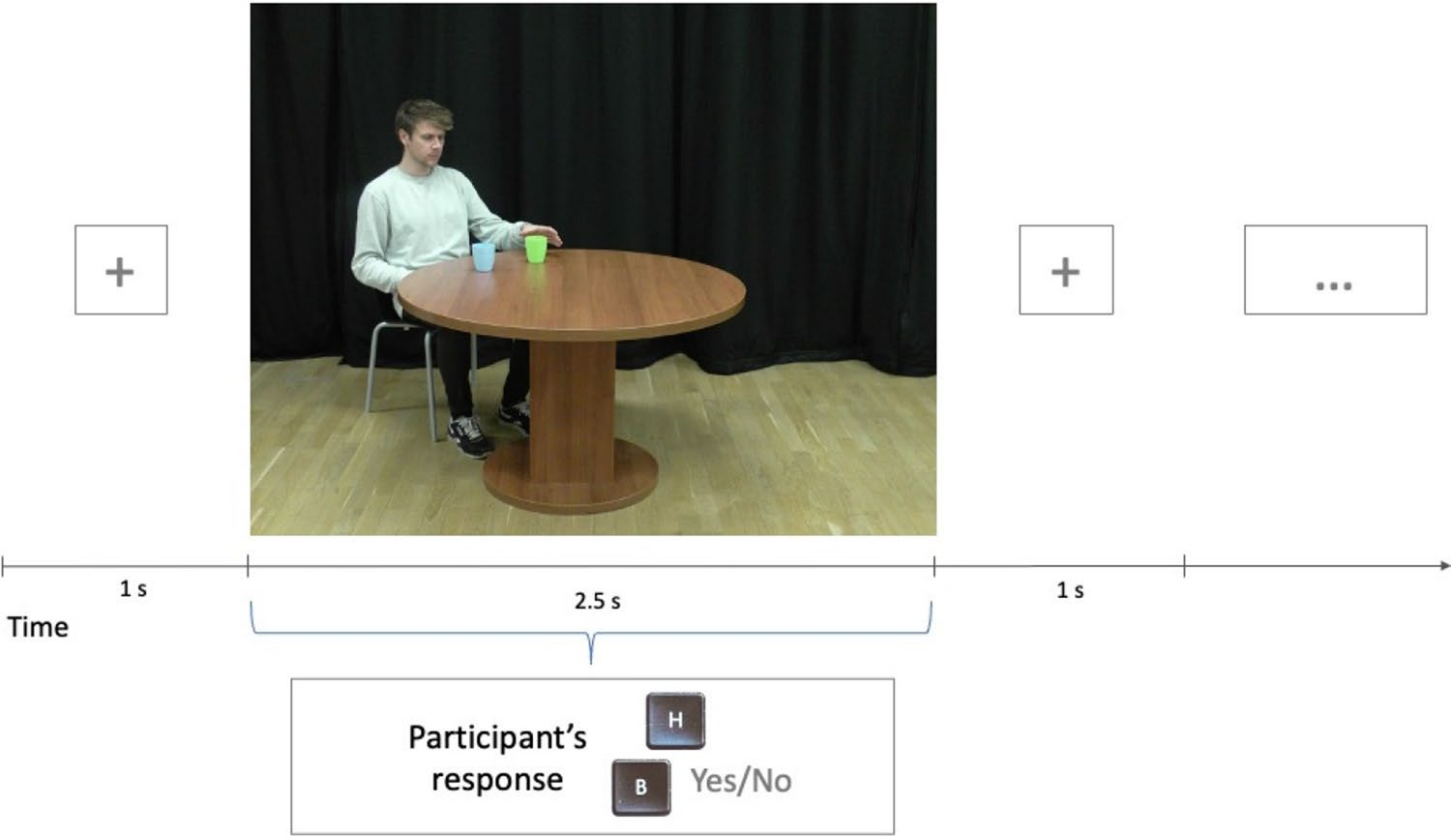
Trial structure in Experiment 1a. Each block of 32 trials started with a question about one of the cups (blue or green), specifying the target cup in that block. The question was presented only once at the start of each block. Stimuli pictures depicting one of the two conditions and different angles were intermixed and presented in random order after the question. Participants used the keys “B” and “H” on the keyboard to respond. Each participant was assigned one of two key layouts: either the “B” key was assigned to the “yes” answer, and the “H” key was assigned to the “no” answer; or the “B” key was assigned to the “no” answer, while the “H” key was assigned to the “yes” answer.

Each participant was presented with 448 test trials: 224 in the “Action” condition and 224 in the “No Action” condition, with each angle occurring equally often in both conditions. Each cup was placed on the right and the left side in the stimuli pictures the same number of times. All types of trials were intermixed and presented in random order. Each block of 32 trials started with a question about one of the cups (blue or green), specifying the target cup in that block. Participants were asked about the two cups an equal number of times. Trial structure in Experiment 1a is presented in Fig. 2.

In addition, for the stimuli in the “Action” condition, we had 3 sets of stimuli pictures depicting a very slightly different hand posture during action (three subtle variations of depicted action for each angular disparity). This was done to prevent any possibility that a specific picture would cause any unexpected effects. We intermixed and randomly assigned different pictures between participants. We also introduced 128 extra filler trials for both groups that included pictures showing the model reaching for a cup with the other hand than the one shown to that group. That is, in Group 1, where the model used the right hand to reach for a cup on the right, we intermixed additional pictures of the model reaching with their left hand towards a cup on their left; in Group 2, where the model used the left hand to reach for a cup on the left, we intermixed trials of the model reaching with their right hand towards an object on their right. These trials were added to make the task harder and to prevent participants from always focusing on only one side. These trials were not included in the analyses.

After completing the task, participants were asked to report their age, gender, and the strategy that they had used in the task, and they were asked to indicate whether they had experienced any technical difficulties during the task. Participants were also asked to fill out two questionnaires: the Interpersonal reactivity index^23^ and the Autism Spectrum Quotient^24^.

#### Data analyses

Accuracy data were analyzed based on the percentage of correct responses in each condition and for each angle for each participant. Reaction time data analyses included only reaction times (RTs) of correct responses. RTs of correct trials that were above or below 2.5 standard deviations from the mean of the correct RTs for each participant in each of the two conditions or faster than 200 ms were excluded from the RT analyses.

The accuracy and RT data of Group 1 and Group 2 were analyzed together because we did not have any hypotheses concerning differences between seeing a left versus right-hand action. To verify that the hand used by the model did not make a difference, we conducted a mixed effects ANOVA with the between-participant factor Group (“1”, “2”) and within-participant factors Condition (“Action”, “No Action”) and Angle (45, 90, 135, 180, 225, 270, 315) on the RT data. Given that the analyses showed neither a significant main effect of Group nor a significant interaction of Group and Condition, we report the results of analyses that encompass both groups. For detailed results of these analyses, see TableS1 in Supplementary Information.

A within-participants ANOVA with the factors Condition (“Action” and “No Action”) and Angle (45, 90, 135, 180, 225, 270, 315) was performed on the RT data of both groups. If the sphericity assumption was violated, the Greenhouse–Geisser correction was applied to the degrees of freedom. We also performed Bayesian ANOVA on the RT data using default prior scales^25^.

Generalized Linear Mixed Effects Modeling (GLMM) was used to examine the binary response data (using a logistic link). For this analysis, condition (“Action”, “No Action”), and Angle (45, 90, 135, 180, 225, 270, 315) were entered as fixed effects along with their interactions, and a random intercept was used across participants. The significance of fixed effects was assessed via likelihood ratio tests of nested models. For the questionnaire data analyses, we calculated Kendall’s tau correlation coefficients of both RT and accuracy data with the final scores of the IRI and AQ measures, and separately with the final scores of the perspective taking (PT) scale in the IRI.

Data analyses were conducted in R software^26^ using packages “ez”^27^, lme4^28^, BayesFactor (v. 0.9.12)^29^ and “effect size”^30^. Figures were produced using the package “ggplot2”^31^.

### Results

#### Accuracy

Likelihood ratio tests of GLMM analysis of the accuracy data revealed a statistically significant effect of condition, χ^2^(1) = 9.09, *p* = 0.003. Participants were more accurate in the “Action” condition compared to the “No Action” condition. The effect of Angle was also significant, χ^2^(5) = 53.25, *p* < 0.001. Participants were less accurate when presented with stimuli pictures with higher angular disparities between them and the model in the pictures. The model that included the interaction between Condition and Angle did not fit the data significantly better compared to the model without Condition and Angle interaction as a fixed effect, χ^2^(6) = 3.77, *p* = 0.71. The model comparisons and estimated effects are presented in Tables S3 and S4 in Supplementary Material.

**Figure 3:**
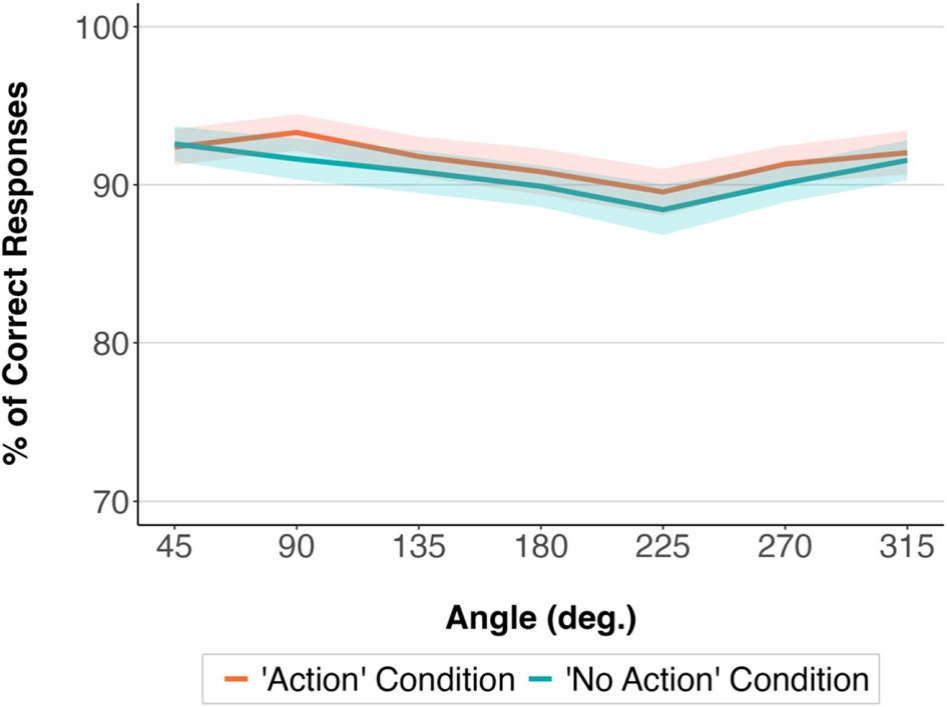
Mean accuracy rate in the “Action” and “No Action” condition at different angular disparities in Experiment 1a. The shaded areas around the means represent 95% confidence intervals calculated after removing the between-participants variability^32^.

The results are shown in Fig. 3.

#### Reaction times

The 2 by 7 within-participants ANOVA with the factors Condition (“Action”, “No Action”) and Angle (45°, 90°, 135°, 180°, 225°, 270°, and 315°) showed a significant effect of Condition, *F* (1, 77) = 9.49, *p* = 0.003, partial eta squared = 0.11, with RTs being faster in the “Action” condition compared to the “No Action” condition. The effect of Angle was also significant, *F* (3, 221) = 11.60, *p* < 0.001, partial eta squared = 0.13. The participants were slower at higher angular disparities between them and the model in the pictures. The interaction between Condition and Angle was not significant, *F* (5, 404) = 0.32, *p* = 0.906.

Paired t-test post-hoc comparisons (with applied Bonferroni correction for multiple pairwise comparisons) showed that at the lowest, 45° and 315°, angular disparities, participants were significantly faster compared to each of the higher angular disparities (including 90° and 270°) on both the left and right side (*p* < 0.02).

Further, Bayesian analyses comparing the full model (including the effects of Condition, Angle, and Condition and Angle interaction) with the models without each of these effects and the interaction provided evidence in favour of keeping Condition (Bayes factor, BF_log10_ = − 0.38) and Angle (BF_log10_ = − 46.80) in the model. Importantly, the analyses provided decisive evidence in favour of removing the Condition and Angle interaction from the model (BF_log10_ = 6.70), showing the absence of the interaction effect.

**Figure 4:**
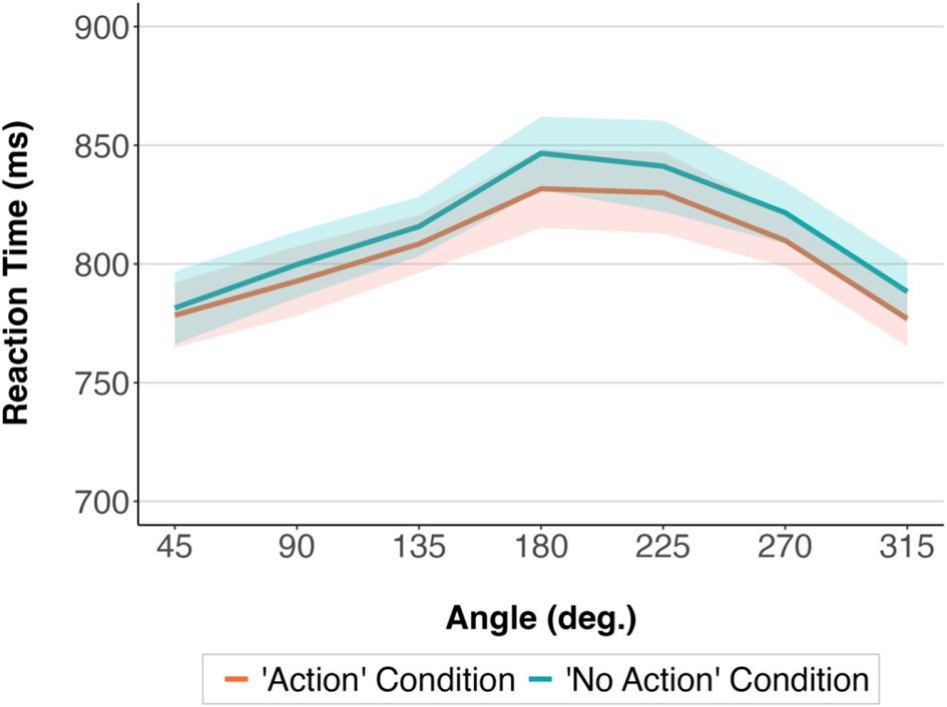
Mean reaction times in the “Action” and “No Action” condition at different angular disparities in Experiment 1a. The shaded areas around the means represent 95% confidence intervals calculated after removing the between-participants variability^32^.

The results are shown in Fig. 4.

#### ‘Yes’ and ‘*N*o’ answers

Adding Answer Type (‘Yes’, ‘No’) as a factor to the within participants ANOVA revealed a significant effect of Answer Type in the RT data, *F* (1, 77) = 82.78, *p* < 0.001, partial eta squared = 0.53. The participants were faster when the correct answer was ‘Yes’ (*M* = 766 ms; *SD* = 279 ms) compared to when it was ‘No’ (*M* = 838 ms; *SD* = 292 ms). However, none of the interactions with the other factors (Angle or Condition) were significant (*p* > 0.43).

Likelihood ratio tests of GLMM analysis of the accuracy data with added fixed effect Answer Type revealed a statistically significant effect of Answer Type, χ^2^(1) = 15.95, *p* < 0.001. Participants were more accurate when the required answer was ‘Yes’ (*M* = 92%, *SD* = 11%) compared to ‘No’ (*M* = 91%, *SD* = 12%). Adding any interaction effects with other factors to the model did not significantly improve its fit (*p* > 0.76).

#### Questionnaires

The results showed a significant positive correlation between participants' RTs and the PT scale of the IRI in Experiment 1a (*τ* = 0.17, *p* = 0.04). This may indicate that participants with higher scores on the PT scale were also slower in perspective taking. Other correlations between the IRI and AQ scores and the RT and accuracy data were not significant (presented in Supplementary Material, section 4.

### Discussion

The results of Experiment 1a showed that participants were both more accurate and faster in the “Action” condition compared to the “No Action” condition. This indicates that observing an object-directed action speeded up judgments about the object’s location from another’s perspective. The results also showed the expected main effect of Angle. Regardless of the condition, it was harder for participants to take the perspective of the model with increased angular disparity. This pattern has been consistently found in previous studies^4,5^ and reflects mental own-body rotation when taking another’s perspective.

Importantly, there was no significant interaction between Angle and Condition in accuracy and reaction time data. This indicates that the facilitatory effect of observing a goal-directed action was independent of mental own-body rotation. Seeing others acting facilitated judgments about the position of objects relative to their body, while it did not affect the ease with which mental own-body rotation was performed. The general facilitatory effect of action observation could be due to action simulation processes that shift attention toward the object to be grasped. Specifically, it has been proposed that observing actions triggers a simulation process that shifts attention to regions of space where the consequences of the action are likely to unfold^33,34^. However, in the “No action” condition, the hand was far from the object (resting on the model’s legs), so we cannot exclude the possibility that the mere presence of a hand near the object, rather than seeing a goal-directed action, facilitated judgments about the object’s location relative to the model’s body. Therefore, in Experiment 2, we aimed to replicate the effects of Experiment 1a with stimuli that controlled for the hand’s vicinity to the cup to be judged.

Before doing so, in Experiment 1b, we aimed to replicate the results by Kessler and Thompson (Experiment 1 vs. Experiment 2)^4^, showing that the presence of a human figure that provides a possibility for bodily mapping has a facilitatory effect on perspective taking, especially for larger angular disparities.

## Experiment 1b

**Figure 5:**
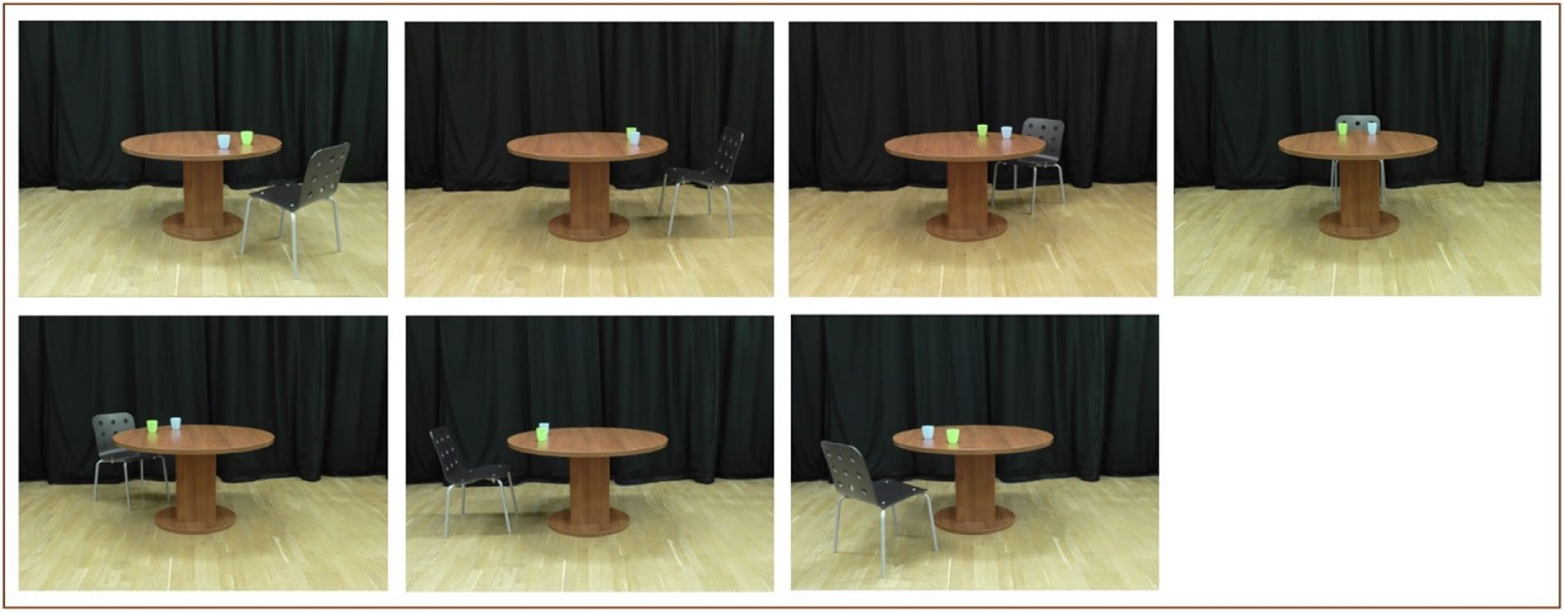
Stimuli pictures in the “Person Absent” condition in experiment 1b.

In Experiment 1b, we introduced a condition in which no person was present, and instead, there was a chair in the scene. In this “Person Absent” condition, we presented participants with stimuli pictures showing a chair at a round table at 7 different angles (45°, 90°, 135°, 180°, 225°, 270°, and 315°) (see Fig. 5). We asked participants to indicate the relative position of one of the two cups from the perspective of the chair. At the beginning of the experiment, it was clear to participants that making left/right judgments from the chair's perspective would mean taking into account the direction the chair was facing. For example, in Fig. 5, the green cup is always on the right from the perspective of the chair.

The results of this condition were compared to the results of the “No Action” condition of Experiment 1a.

### Methods

#### Participants

To be able to compare the results to the ones obtained in the “No Action” condition of Experiment 1a, we aimed to recruit the same number of participants. As in Experiment 1a, there were two groups of participants (Note: in the preregistration of the experiment, the two groups of participants are pre-registered as two experiments, however, to report the results more succinctly, we chose to use the term Group here). We recruited 40 participants in Group 1 (mean age = 24.13, *SD* = 4.76) and 40 Participants in Group 2 (mean age = 23.45, *SD* = 4.72) (See section *Participants* under “Experiment 1a” for sample size calculations). Participants were recruited using the same online platform for participant recruitment (Prolific, www.prolific.co). Participants had normal or corrected-to-normal vision, were right-handed, and were between 18 and 35 years old.

Participants with an accuracy rate of two standard deviations below the mean accuracy rate of all participants in each group were excluded from the data analyses.

The study was approved by the local Ethical committee and was conducted according to the Helsinki Declaration^20^. Before the experiment, each participant was provided with information about the study and was asked to give consent to participate. Participants were paid 4.60 GBP for their participation.

#### Procedure and stimuli

The procedure was largely the same as in Experiment 1a. In Group 1, the participants were asked if one of the cups (blue/green) is on the right side from the perspective of the chair (“In the following trials, is the blue cup on the right of the chair?”, “In the following trials, is the green cup on the right of the chair?”). In Group 2, the participants were asked if a cup is on the left side of the chair in the pictures (“In the following trials, is the blue cup on the left of the chair?”, “In the following trials, is the green cup on the left of the chair?”). Participants were asked to respond “yes” or “no”, and “B” and “H” keys on the keyboard were assigned to each answer. The assignment of one of two key layout variations was randomly set for each participant: in Layout 1, the “B” key on the keyboard was assigned to the “yes” answer, and the “H” key was assigned to the “no” answer; in Layout 2, the “B” key was assigned to the “no” answer, while the “H” key was assigned to the “yes” answer.

In total, each participant was presented with 224 trials. 32 trials were presented for each angle. Trials for all seven angles were intermixed and presented in random order.

#### Data analyses

As in Experiment 1a, we analyzed the data of both groups of participants together. We compared the “Person Absent” condition to the data obtained in the “No Action” condition in Experiment 1a with the model sitting still with both of their hands on the lap. Here, we refer to it as the “Person Present—No Action” Condition. The total number of participants was 158 (80 participants in the “Person Absent” condition; 78 participants in the “Person Present—No Action” condition). We performed a mixed effects ANOVA with the between-participant factor Condition (“Person Absent”, “Person Present—No Action”) and the within-participant factor Angle (45, 90, 135, 180, 225, 270, 315) on the RT data. If the sphericity assumption was violated, the Greenhouse–Geisser correction was applied to the degrees of freedom. We also performed Bayesian ANOVA on the RT data using default prior scales^25^. Generalized Linear Mixed Effects Modeling (GLMM) was used to examine the binary response data (using a logistic link). For this analysis, condition (“Person Absent”, “Person Present—No Action”), and Angle (45, 90, 135, 180, 225, 270, 315) were entered as fixed effects along with their interactions, and a random intercept was used across participants. The significance of fixed effects was assessed via likelihood ratio tests of nested models.

### Results

#### Accuracy

**Figure 6:**
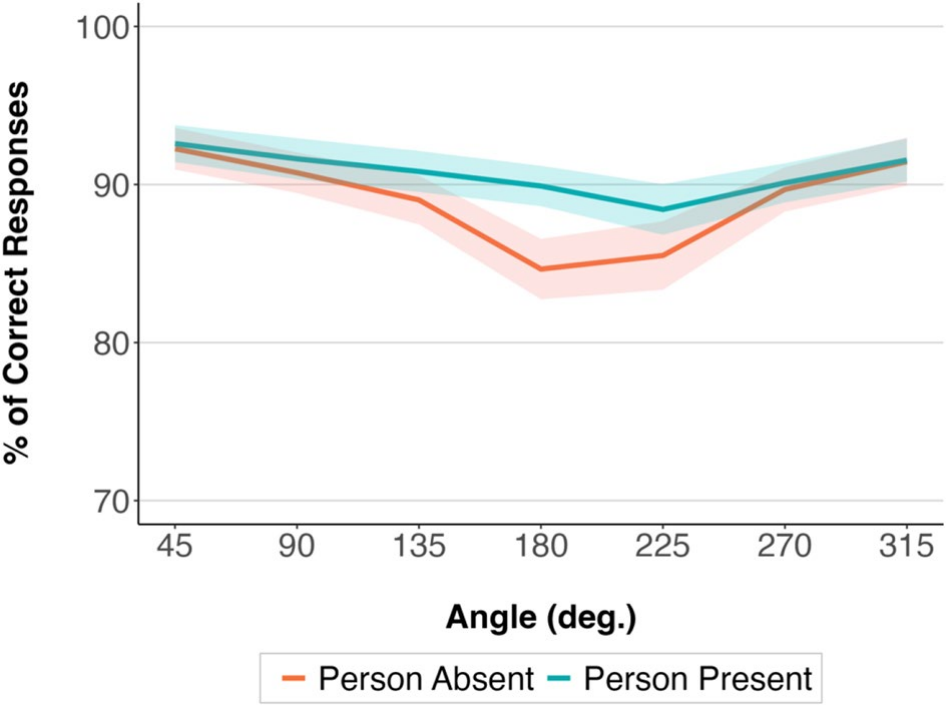
Mean accuracy rate in the “Person Present—No Action” and “Person Absent” condition at different angular disparities in Experiment 1b. The shaded areas around the means represent 95% confidence intervals calculated after removing the between-participants variability^32^.

Likelihood ratio tests of GLMM analysis of the accuracy data showed a statistically significant effect of Angle, χ^2^(5) = 155.58, *p* = < 0.001, and a significant interaction between Angle and Condition, χ^2^(6) = 21.92, *p* = 0.001. The model comparisons and estimated effects are presented in Tables S5 and S6 in Supplementary Material. The results are illustrated in Fig. 6.

#### Reaction Times

The 2 by 7 mixed-effects ANOVA with the between-participant factor Condition (“Person Absent”, “Person Present—No Action”) and within-participant factor Angle (45°, 90°, 135°, 180°, 225°, 270°, 315°) showed a significant effect of Condition, *F* (1, 156) = 36.11, *p* < 0.001, partial eta squared = 0.19, and a significant effect of Angle, *F* (4, 599) = 47.85, *p* < 0.001, partial eta squared = 0.24. The interaction between Condition and Angle, *F* (4, 599) = 11.13, *p* < 0.001, partial eta squared = 0.07, was also significant.

Welch two-sample t-test post-hoc comparisons (with applied Bonferroni correction for multiple pairwise comparisons) showed that the differences between the “Person Absent” and “Person Present—No Action” conditions were present at each angular disparity (*p* < 0.001). Paired t-test post-hoc comparisons (with applied Bonferroni correction for multiple pairwise comparisons) showed that at the lowest, 45° and 315°, angular disparities, participants were significantly faster compared to each of the higher angular disparities (including 90° and 270°) on both the left and right sides in both “Person Absent” (*p* < 0.001) and “Person Present—No Action” (*p* < 0.023) conditions.

Further, Bayesian analyses comparing the full model (including the effects of Condition, Angle, and Condition and Angle interaction) with the models without each of these effects and the interaction provided evidence in favour of keeping Condition (BF_log10_ = − 5.86), Angle (BF_log10_ = − 136.76) and the Condition and Angle interaction (BF_log10_ = − 24.13) in the model.

**Figure 7:**
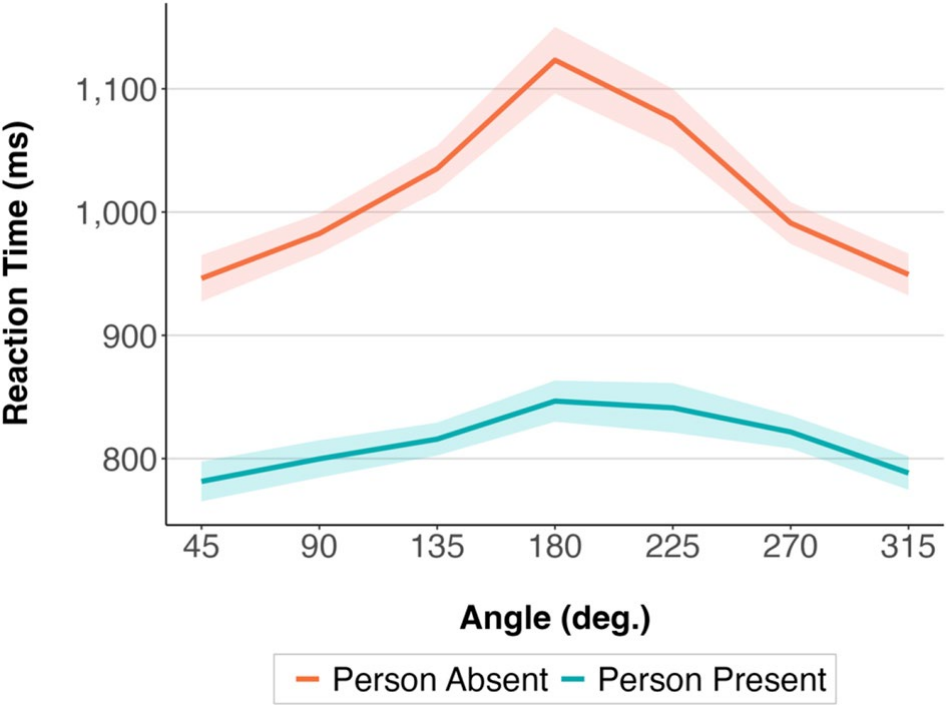
Mean reaction times in the “Person Present—No Action” and “Person Absent” condition at different angular disparities in Experiment 1b. The shaded areas around the means represent 95% confidence intervals calculated after removing the between-participants variability^32^.

The results are illustrated in Fig. 7.

#### ‘Yes’ and ‘No’ answers

Adding Answer Type (‘Yes’, ‘No’) as a factor to the mixed effects ANOVA revealed a significant effect of Answer Type, *F* (1, 156) = 149.00, *p* < 0.001, partial eta squared = 0.49. The participants were faster when the correct answer was ‘Yes’ (*M* = 863 ms; *SD* = 349 ms) compared to when it was ‘No’ (*M* = 942 ms; *SD* = 359 ms). However, none of the interactions with the other factors (Angle or Condition) were significant (*p* > 0.17).

Likelihood ratio tests of GLMM analysis of the accuracy data with added fixed effect Answer Type revealed a statistically significant effect of Answer Type, χ^2^(1) = 16.76, *p* < 0.001. Participants were more accurate when the required answer was ‘Yes’ (*M* = 91%, *SD* = 12%) compared to ‘No’ (*M* = 89%, *SD* = 13%). Adding interaction effects with other factors to the model did not significantly improve its fit (*p* > 0.34).

### Discussion

In Experiment 1b, we investigated differences in reaction time and accuracy patterns between taking the perspective of a person and taking the perspective of an empty chair positioned at the same angles. We aimed to replicate the findings of the experiments conducted by Kessler and Thomson (Experiment 1 vs. 2)^4^, which showed that taking the perspective of a person is faster than taking the perspective of a chair and that these differences are larger at higher angular disparities. Our results replicated these findings and showed that taking the perspective of a person is much faster than taking the perspective of an empty chair. Importantly, our results also showed that these differences were the highest at the highest angular disparities. This pattern was present both in the reaction time and in the accuracy data. This indicates that having a possibility for bodily mapping is crucial when taking a perspective at higher angular disparities between us and a place from which we are asked to take the perspective. Having a human figure as a reference and being able to mentally align our bodily axes with theirs could lessen the cognitive load that is put on imagery processes at the highest angular disparities. However, given that we relied on a between-participants design we cannot rule out the possibility that there may be differences in overall reaction speed between the two groups of participants. Future research should aim to replicate the results using a within-participants design.

## Experiment 2

In Experiment 1a, we found facilitatory effects of observing another’s action on participants’ RTs and accuracy rates. However, in the “Action” condition, the hand was closer to the cup, possibly cueing participants’ attention to the cup on that side, while in the “No Action” condition, both hands were further away from the cups. In Experiment 2, we controlled for this difference by showing participants pictures in the “No Action” condition where the model placed one hand on the table next to one of the cups. This made the “Action” and “No Action” conditions visually much more similar. As in Experiment 1a, in Experiment 2, we divided participants into two groups (Note: in the preregistration of the experiment, the two groups of participants are pre-registered as two experiments, however, to report the results more succinctly, we chose to use the term Group here). For Group 1, in the “No Action” condition, the model’s right hand was placed towards the cup on their right. In Group 2, the model’s left hand was placed on their left side towards the cup on their left (see Fig. 1b for examples of stimuli pictures). The task was the same as in Experiment 1a. In Group 1, the participants were asked if the blue/green cup is on the right of the person (“In the following trials, is the blue cup on the right of the person?”, “In the following trials, is the green cup on the right of the person?”); in Group 2, the participants were asked if the blue/green cup is on the left side of the person (“In the following trials, is the blue cup on the left of the person?”, “In the following trials, is the green cup on the left of the person?”). The stimuli pictures in the “Action” condition were the same as in Experiment 1a.

### Methods

#### Participants

Using the same online platform for participant recruitment (Prolific, www.prolific.co) as in Experiment 1a, we recruited 40 participants in Group 1 (mean age = 24.75, *SD* = 4.50) and 40 Participants in Group 2 (mean age = 24.89, *SD* = 4.80). We recruited participants who had normal or corrected to normal vision, were right-handed, and were between 18 and 35 years old. The sample size calculation was based on the same power analyses as in Experiment 1a.

As in Experiment 1a, participants with an accuracy rate of two standard deviations below the mean accuracy rate of all participants in each experiment were excluded from the data analyses. One participant indicated that they had been diagnosed with autism spectrum disorder. The data of this participant was not included in the analyses because ASD may come with differences in the use of mental own body rotation^9^. In total, the data of 76 participants were analyzed in Experiment 2.

The study was approved by the local Ethics committee (Psychological Research Ethics Board PREBO) and was conducted according to the Helsinki Declaration^21^. Before the experiment, each participant was provided with information about the study and was asked to give informed consent. Participants were paid 6 GBP for their participation.

#### Design

As in Experiment 1a, the experiment had a within-participant design with the factors Condition (“Action” and “No Action”) and Angle (seven different angles: 45°, 90°, 135°, 180°, 225°, 270°, 315°)

#### Procedure and stimuli

The procedure in Experiment 2 was the same as the procedure in Experiment 1a, including the presentation of filler trials. The only difference was that the stimuli in the “No Action” condition showed the model with one hand placed on the table next to one of the objects. In Group 1 in Experiment 2, in the “No Action” condition, the model’s right hand was placed towards the cup on their right. In Group 2, the model’s left hand was placed on their left side towards the cup on their left (see Fig. 1b).

#### Data analyses

As in Experiment 1a, the accuracy and RT data of Group 1 and Group 2 were analyzed together because we did not have any hypotheses concerning differences between seeing a left versus right-hand action. To verify that the hand used by the model did not make a difference, we conducted a mixed effects ANOVA with the between-participant factor Group (“1”; “2”) and within-participant factors Condition (“Action”, “No Action”) and Angle (45, 90, 135, 180, 225, 270, 315) on the RT data. Given that the analyses showed neither a significant main effect of Group nor a significant interaction of Group and Condition, we report the results of analyses that encompass both groups. For detailed results of these analyses, see Table S2 in Supplementary Information.

A within-participants ANOVA with the factors Condition (“Action” and “No Action”) and Angle (45, 90, 135, 180, 225, 270, 315) was performed on the RT data. If the sphericity assumption was violated, the Greenhouse–Geisser correction was applied to the degrees of freedom. We also performed Bayesian ANOVA on the RT data using default prior scales^25^.Generalized Linear Mixed Effects Modeling (GLMM) was used to examine the binary response data (using a logistic link). For this analysis, condition (“Action”, “No Action”), and Angle (45, 90, 135, 180, 225, 270, 315) were entered as fixed effects along with their interactions, and a random intercept was used across participants. The significance of fixed effects was assessed via likelihood ratio tests of nested models.

For the questionnaire data analyses, we calculated Kendall’s tau correlation coefficients of both RT and accuracy data with the final scores of the IRI and AQ measures, and separately with the final scores of the perspective taking (PT) scale in the IRI.

### Results

#### Accuracy

Likelihood ratio tests of GLMM analysis of the accuracy data revealed a statistically significant effect of Angle, χ^2^(5) = 78.03, *p* < 0.001. Participants were less accurate at the higher angular disparities between them and the model in the stimuli pictures (% correct, 45 deg.: *M* = 94.70, *SD* = 7.29; 90 deg.: *M* = 94.12, *SD* = 7.31; 135 deg.: *M* = 93.09, *SD* = 7.51; 180 deg.: *M* = 93.57, *SD* = 7.72; 225 deg.: *M* = 91.82, *SD* = 7.46; 270 deg.: *M* = 93.98, *SD* = 7.60; 315 deg.: *M* = 95.66, *SD* = 5.11). The effect of Condition, χ^2^(1) = 0.90, *p* = 0.34, and the interaction between Condition and Angle, χ^2^(6) = 5.38, *p* = 0.50, were not significant. Model comparisons and estimated effects are presented in Tables S7 and S8 in Supplementary Material.

#### Reaction times

The 2 by 7 within-participants ANOVA with the factors Condition (“Action”, “No Action”) and Angle (45, 90, 135, 180, 225, 270, 315 degrees) showed a significant effect of Condition, *F* (1, 75) = 4.75, *p* = 0.032, partial eta squared = 0.06, and a significant effect of Angle, *F* (3, 241) = 10.64, *p* < 0.001, partial eta squared = 0.12. The interaction between Condition and Angle was not significant, *F* (5, 377) = 1.64, *p* = 0.147.

Paired t-test post-hoc comparisons (with applied Bonferroni correction for multiple pairwise comparisons) showed that at the lowest, 45° and 315°, angular disparities, participants were significantly faster compared to each of the higher angular disparities (including 90° and 270°) on both the left and right side (*p* < 0.006).Bayesian analyses comparing the full model (including the effects of Condition, Angle, and Condition and Angle interaction) with the models without each of these effects and the interaction provided evidence in favour of keeping Angle in the model (BF_log10_ = − 38.89). The analyses provided decisive evidence in favour of removing the Condition and Angle interaction (BF_log10_ = 5.12) and indecisive evidence for removing Condition (BF_log10_ = 0.40) from the model.

**Figure 8:**
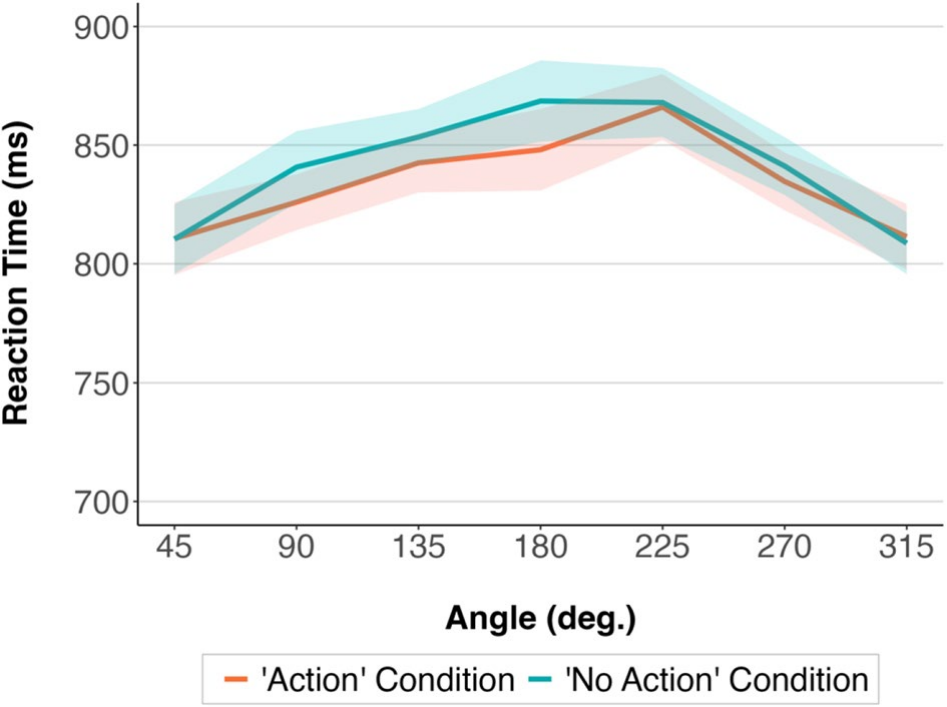
Mean reaction times in the “Action” and “No Action” condition at different angular disparities in Experiment 2. The shaded areas around the means represent 95% confidence intervals calculated after removing the between-participants variability^32^.

The results are shown in Fig. 8.

#### ‘Yes’ and ‘No’ answers

Adding Answer Type (‘Yes’, ‘No’) as a within-participant factor to ANOVA revealed a significant effect of Answer Type, *F* (1, 75) = 96.80, *p* < 0.001, partial eta squared = 0.56. The participants were faster when the correct answer was ‘Yes’ (*M* = 796 ms; *SD* = 290 ms) compared to when it was ‘No’ (*M* = 863 ms; *SD* = 307 ms). However, none of the interactions with the other factors (Angle or Condition) were significant (*p* > 0.33).

Likelihood ratio tests of GLMM analysis of the accuracy data with added fixed effect Answer Type revealed a statistically significant effect of Answer Type, χ^2^(1) = 13.58, *p* < 0.001. Participants were more accurate when the required answer was ‘Yes’ (*M* = 94%, *SD* = 9%) compared to ‘No’ (*M* = 93%, *SD* = 10%). Adding any interaction effects with other factors to the model did not significantly improve its fit (*p* > 0.32).

#### Questionnaires

The results showed a significant positive correlation between participants' RTs and the total scale of the IRI (*τ* = 0.24, *p* < 0.01). The positive correlation indicates that participants with higher overall scores were also slower in perspective taking. Other correlations with the RT and accuracy data were not significant (presented in Supplementary Material, section 4.

### Discussion

Replicating the results of Experiment 1a, participants were faster to respond in the “Action” condition compared to the “No Action” condition. Also, with increased angular disparities between the participants and the model, reaction times were slower, and accuracy rates were lower, showing the same pattern as in Experiment 1a. As in Experiment 1a, the interaction between Condition and Angle was not significant, indicating that mental own-body rotation and the facilitation effects of action observation are independent. The accuracy rate did not show a significant effect of Condition in Experiment 2, while this effect was significant in Experiment 1a.

The results of the correlation analyses between the IRI and AQ scores and participants’ response time and accuracy measures do not seem to be fully conclusive. As in Experiment 1 a, we did not find any significant correlations between participants' performance and the AQ scores. For the IRI, participants' overall score correlated with their RTs, while in Experiment 1a we found a significant correlation only with the PT scale of the IRI. Overall, one could conclude that participants with a higher inclination to take others' perspective acted more slowly, but the reasons for this pattern remain unclear.

## General discussion

In the current study, we investigated whether action observation facilitates perspective taking and whether and how this facilitation might interact with mental own-body rotation during perspective taking. We also aimed to replicate previous research findings showing the importance of the presence of another person and the possible role of bodily mapping in perspective taking^4^.

In Experiments 1a and 2, we presented participants with an “Action” and a “No Action” condition. In Experiment 1a, in the “No Action” condition, the model in the stimuli pictures had both hands on their lap. In Experiment 2, in the “No Action” condition, the model had one of their hands placed on the table towards the cup on the same side to have a similar distance to the cup as in the “Action” condition. Experiments 1a and 2 showed that seeing an object-directed action facilitated performance. Participants were faster in the “Action” condition compared to the “No Action” condition in both experiments. In Experiment 1a, participants were also more accurate in the “Action” condition compared to the “No Action” condition.

As expected, we found a significant main effect of Angle in the reaction time and accuracy data both in Experiment 1a and Experiment 2. The reaction times increased, and the accuracy rates decreased with the higher angular disparity between participants and the model in the stimuli pictures. This pattern reflects the effort required to mentally rotate one’s body into another’s position^4,5,35,36^.

However, the interaction between Angle (45°, 90°, 135°, 180°, 225°, 270°, 315°) and Condition (“Action”, “No Action”) was not significant. This indicates that the facilitatory effects of seeing another person acting towards an object were consistent throughout different levels of mental own-body rotation during perspective taking. Importantly, spatial incongruency effects do not seem to modulate this facilitation. At the opposite angular disparities (135°, 180°, and 225°), when the perspectives of participants and the model are mainly incongruent, the more salient object from the participant’s perspective is on the opposite side compared to the model’s perspective. Orienting attention towards one of the objects and making one of them more salient could interfere with participants’ judgments from another’s perspective at these angular disparities. However, we did not find that action facilitation effects on perspective taking were less pronounced at the opposite angular disparities, indicating that participants were faster not only in locating the object when it was being acted upon but also in taking the perspective of the person.

The underlying mechanism behind this facilitation could be action simulation processes that involve anatomical mapping. Anatomical mapping means that action simulation runs on effector-specific maps^37^. These processes may facilitate responses related to a target in the regions of space where the outcome of the action is likely to unfold^33,34^. Findings indicating similar mechanisms were obtained in a study by Mazzarella et al.^11^. First, they showed that when participants saw a photograph of a person sitting across a table and acting towards one of the two objects on the table, the tendency to take the person’s perspective increased compared to when seeing that person sitting still. Seeing the person gazing at the object, a strong cue for orienting another’s attention^38,39^, did not increase the tendency to take their perspective. Strikingly, when the task was shifted from a perspective taking task to a target detection task with exactly the same stimuli, the gaze cueing had a facilitatory effect for target detection while seeing the action towards the same object did not. This indicates that when we see a person acting toward an object, qualitatively different processes that involve anatomical mapping are initiated compared to other directional cues that could orient attention toward the same object. Our findings further support this possibility and indicate that the angular disparity between participants and the perceived actor leaves this anatomical mapping unaffected.

However, in the current study, we investigated only the trials in the “Action” condition in which participants determined the relative location of the cup that the model was acting toward. Therefore, we cannot rule out the possibility that action simulation processes may also facilitate response speed through a more general heightened arousal of the action system^33,34^. This could lead to overall faster responses in the “Action” condition compared to the “No Action condition”. This possibility must be addressed in future research.

In Experiment 1b, we investigated if the possibility for bodily mapping that is present when taking the perspective of a person facilitates perspective taking. The results showed a facilitatory effect when taking the perspective of a person compared to when taking the perspective of an empty chair, both in the reaction time and in the accuracy data. The interaction between condition (“Person Absent” and “Person Present”) and angular disparity was also significant both in the reaction time and in the accuracy data. The results showed the largest differences between the conditions at the highest angular disparities between the participants and the model. Mentally mapping one’s bodily axes onto another’s could significantly lessen the cognitive load necessary to sustain and manipulate mental images of oneself in another place and at another orientation when more mental own-body transformation is needed.

### Limitations and directions for future research

In the current study, in the “Action” condition, we focused on the trials in which participants determined the relative location of the cup that the model was acting toward. However, it is important to investigate if action affects how easy it is to determine the relative location of the action’s target object or if action observation facilitates response times during perspective taking through a more general heightened arousal of the action system, leading to faster responses^33,34^. We hope to disentangle these two possibilities in future research.

The current study did not include a control task that would evidently exclude mental-own body transformation, for example, a 0-degree angular disparity or an object detection task. It may be that participants perform some mental own-body transformation even at the lowest angular disparities, at 45° and 315°, even though the task could be solved by direct visual matching. A control condition that would eliminate mental own-body transformation would be helpful to determine whether action observation has a facilitatory effect even when no mental own body transformation is involved.

Further, we showed participants an object-directed grasping movement. However, we often need to take the perspective of a person when they are performing actions that are not aimed at any objects—for example, when learning to dance or practicing specific movements in sports. Future experimental work may wish to investigate how observing these types of actions interacts with perspective taking.

In addition, more experimental work is needed to investigate the mechanisms behind the strong facilitation when taking the perspective of a human figure compared to an inanimate object that was shown in Experiment 1b and in previous research^4^. It is important to explore what kind of a figure or an object would induce similar facilitation effects. Some important features of a figure could include its sidedness, anthropomorphism, animacy, and humanlike appearance^13,40^. It would be important to see which of these dimensions and what degrees of them are sufficient to induce facilitation in perspective taking.

## Conclusions

The current experimental work showed that seeing a person acting had a facilitatory effect when taking their perspective. Further, action observation seemed to facilitate perspective taking similarly across different angular disparities and independently of the degree of mental own-body transformation. The processes whereby action observation facilitated perspective taking likely involve anatomical mapping that is independent of the congruency between the participants’ perspective and the model’s perspective.

Further, taking the perspective of a person was faster and more accurate than taking the perspective of an inanimate object (a chair). The differences between the “Person Present” and “Person Absent” conditions were the largest at the highest angular disparities between the participants and the model in the stimuli pictures. Being able to perform bodily mapping when taking a person’s perspective could have helped reduce the cognitive load that is put on imagery processes at the highest angular disparities.

### Supplementary Information

Supplementary Information.

## Data Availability

Data collected and analyzed during this study are available at: https://osf.io/bhk64/. Experiments 1b and 2 were pre-registered in the Open Science Framework (osf.io). The pre-registration for Experiment 1b can be found at https://osf.io/hsw37. The pre-registration for Experiment 2 can be found at https://osf.io/qyf94.
