# Supplementary material for: The influence of another’s actions and presence on perspective taking
Source: Sci Rep. 2024 Feb 29;14:4971. doi: 10.1038/s41598-024-55200-8 (PMC10904779; doi:10.1038/s41598-024-55200-8)
Supplement: Supplementary file 1 — Supplementary Information. [file 41598_2024_55200_MOESM1_ESM.docx]

**Supplementary Information**

**1. Comparisons Between the Groups Concerning Left and Right Side**

For the data in Experiments 1a and 2, mixed effects ANOVAs with the within-participant factors Condition (“No Action”, “Action”), angle (45, 90, 135, 180, 225, 270, 315 degrees) and the between-participant factor Group (“1” and “2”) were performed on reaction time. Results showed no main effect of the factor Group nor a significant interaction between Group and Condition. The results of the analyses are presented in Supplementary Tables S1 and S2 below.

**Supplementary Table S1**

***The Results of Mixed Effects ANOVA on Rection Times in Experiment 1a***

| Predictor | df_num | df_den | Epsilon | *F* | *p* |
| --- | --- | --- | --- | --- | --- |
| Group | 1 | 76 |  | 1.13 | .292 |
| Condition | 1 | 76 |  | 9.39 | .003 |
| Group x Condition | 1 | 76 |  | 2.15 | .147 |
| Angle | 2.79 | 212.11 | .47 | 11.71 | <.001 |
| Group x Angle | 2.79 | 212.11 | .47 | 2.82 | .044 |
| Angle x Condition | 5.27 | 400.78 | .88 | 0.34 | .895 |
| Group x Angle x Condition | 5.27 | 400.78 | .88 | 1.13 | .344 |

*Note.* df_num indicates degrees of freedom numerator. df_den indicates degrees of freedom denominator. Epsilon indicates the Greenhouse-Geisser multiplier for degrees of freedom, p-values, and degrees of freedom in the table incorporate this correction.

**Supplementary Table S2**

***The Results of Mixed Effects ANOVA on Reaction Times in Experiment 2***

| Predictor | df_num | df_den | Epsilon | *F* | *p* |
| --- | --- | --- | --- | --- | --- |
| Group | 1 | 74 |  | 1.68 | .2 |
| Condition | 1 | 74 |  | 4.81 | .031 |
| Group x Condition | 1 | 74 |  | 1.93 | .169 |
| Angle | 3.13 | 231.78 | .52 | 10.72 | <.001 |
| Group x Angle | 3.13 | 231.78 | .52 | 1.55 | .201 |
| Angle x Condition | 5 | 369.76 | .83 | 1.64 | .15 |
| Group x Angle x Condition | 5 | 369.76 | .83 | 0.61 | .695 |

*Note.* df_num indicates degrees of freedom numerator. df_den indicates degrees of freedom denominator. Epsilon indicates the Greenhouse-Geisser multiplier for degrees of freedom, p-values, and degrees of freedom in the table incorporate this correction.

**2. Results of Generalized Linear Mixed Effects Modeling (GLMM) of Binary Response Data**

Generalized Linear Mixed Effects Modeling (GLMM) was used to examine the binary response data (using a logistic link). For this analysis, condition (“Action”, “No Action” in Experiments 1a and 2; “Person Present”, “Person Absent” in Experiment 1b), and Angle (45, 90, 135, 180, 225, 270, 315) were entered as fixed effects along with their interactions, and a random intercept was used across participants. The significance of fixed effects was assessed via likelihood ratio tests of nested models. You can find the comparisons of the models to the null model (without fixed effects) in Tables S3 (Experiment 1a), S5 (Experiment 1b), S7 (Experiment 2). The effects of the best fitted models that include beta, *SE*, *z* and *p-values* are presented in Tables S4 (Experiment 1a), S6 (Experiment 1b), S8 (Experiment 2).

**Supplementary Table S3**

***Nested Model Comparison via Likelihood Ratio Tests in Experiment 1a***

***Models:***

***M0: Acuraccy ~ 1 + (1 | Participant_number)***

***M1: Acuraccy ~ Condition + (1 | Participant_number)***

***M2: Acuraccy ~ Angle + (1 | Participant_number)***

***M3: Acuraccy ~ Condition + Angle + (1 | Participant_number)***

***M4: Acuraccy ~ Condition + Angle + Condition * Angle + (1 |Participant_number)***

|  | npar | AIC | BIC | logLik | deviance | Chisq | Df | *Pr(>Chisq*) |
| --- | --- | --- | --- | --- | --- | --- | --- | --- |
| M0 | 2 | 18911 | 18928 | -9453.7 | 18907 |  |  |  |
| M1 | 3 | 18904 | 18930 | -9449.2 | 18898 | 9,09 | 1 | .003 |
| M2 | 8 | 18861 | 18929 | -9422.5 | 18845 | 53,25 | 5 | < 0.01 |
| M3 | 9 | 18854 | 18930 | -9418.0 | 18836 | 9,11 | 1 | .003 |
| M4 | 15 | 18862 | 18989 | -9416.1 | 18832 | 3,77 | 6 | 0.71 |

**Supplementary Table S4**

***Generalized Linear Mixed Model Fit by Maximum Likelihood (Laplace Approximation), Using Logistic Link in Experiment 1a.***

***Formula: Accuracy ~ Condition + Angle + (1 | Participant_number) (M3)***

|  | β | *SE* | *z* | *p* |
| --- | --- | --- | --- | --- |
| (Intercept) | 2.94 | 0.12 | 23.69 | < .001 |
| Condition "No Action" | -0.12 | 0.04 | -3.03 | .002 |
| Angle 90 | 0.00 | 0.08 | -0.04 | .97 |
| Angle 135 | -0.17 | 0.08 | -2.25 | .02 |
| Angle 180 | -0.29 | 0.07 | -3.95 | < .001 |
| Angle 225 | -0.46 | 0.07 | -6.29 | < .001 |
| Angle 270 | -0.25 | 0.07 | -3.34 | .001 |
| Angle 315 | -0.10 | 0.08 | -1.35 | .18 |

**Supplementary Table S5**

***Nested Model Comparison via Likelihood Ratio Tests in Experiment 1b***

***Models:***

***M0: Acuraccy ~ 1 + (1 | Participant_number)***

***M1: Acuraccy ~ Condition + (1 | Participant_number)***

***M2: Acuraccy ~ Angle + (1 | Participant_number)***

***M3: Acuraccy ~ Condition + Angle + (1 | Participant_number)***

***M4: Acuraccy ~ Condition + Angle + Condition * Angle + (1 |Participant_number)***

|  | npar | AIC | BIC | logLik | deviance | Chisq | Df | *Pr(>Chisq*) |
| --- | --- | --- | --- | --- | --- | --- | --- | --- |
| M0 | 2 | 21105 | 21122 | -10551 | 21101 |  |  |  |
| M1 | 3 | 21106 | 21132 | -10550 | 21100 | 12.77 | 1 | .258 |
| M2 | 8 | 20960 | 21028 | -10472 | 20944 | 155.58 | 5 | < .001 |
| M3 | 9 | 20961 | 21038 | -10472 | 20943 | 1.28 | 1 | .258 |
| M4 | 15 | 20951 | 21078 | -10461 | 20921 | 21.92 | 6 | .001 |

**Supplementary Table S6**

***Generalized Linear Mixed Model Fit by Maximum Likelihood (Laplace Approximation), Using Logistic Link in Experiment 1b.***

***Formula: Acuraccy ~ Condition + Angle + Condition * Angle + (1 |Participant_number) (M4)***

|  | β | SE | *z* | *p* |
| --- | --- | --- | --- | --- |
| (Intercept) | 2.87 | 0.14 | 20.95 | <.001 |
| ConditionPerson_present | 0.03 | 0.20 | 0.18 | .86 |
| Angle90 | -0.21 | 0.10 | -2.03 | .04 |
| Angle135 | -0.41 | 0.10 | -4.13 | <.001 |
| Angle180 | -0.84 | 0.10 | -8.78 | <.001 |
| Angle225 | -0.76 | 0.10 | -7.93 | <.001 |
| Angle270 | -0.34 | 0.10 | -3.34 | .001 |
| Angle315 | -0.12 | 0.11 | -1.11 | .27 |
| ConditionPerson_present:Angle90 | 0.07 | 0.15 | 0.45 | .65 |
| ConditionPerson_present:Angle135 | 0.16 | 0.15 | 1.12 | .26 |
| ConditionPerson_present:Angle180 | 0.47 | 0.14 | 3.34 | .001 |
| ConditionPerson_present:Angle225 | 0.23 | 0.14 | 1.64 | .10 |
| ConditionPerson_present:Angle270 | 0.00 | 0.15 | -0.02 | .99 |
| ConditionPerson_present:Angle315 | -0.04 | 0.15 | -0.24 | .81 |

**Supplementary Table S7**

***Nested Model Comparison via Likelihood Ratio Tests in Experiment 2***

***Models:***

***M0: Acuraccy ~ 1 + (1 | Participant_number)***

***M1: Acuraccy ~ Condition + (1 | Participant_number)***

***M2: Acuraccy ~ Angle + (1 | Participant_number)***

***M3: Acuraccy ~ Condition + Angle + (1 | Participant_number)***

***M4: Acuraccy ~ Condition + Angle + Condition * Angle + (1 |Participant_number)***

|  | npar | AIC | BIC | logLik | deviance | Chisq | Df | *Pr(>Chisq*) |
| --- | --- | --- | --- | --- | --- | --- | --- | --- |
| M0 | 2 | 14380 | 14397 | -7188.3 | 14376 |  |  |  |
| M1 | 3 | 14382 | 14407 | -7187.8 | 14376 | 0.90 | 1 | .34 |
| M2 | 8 | 14314 | 14381 | -7148.8 | 14298 | 78.03 | 5 | < .001 |
| M3 | 9 | 14315 | 14391 | -7148.3 | 14297 | 0.91 | 1 | .34 |
| M4 | 15 | 14321 | 14448 | -7145.7 | 14291 | 5.38 | 6 | .50 |

**Supplementary Table S8**

***Generalized Linear Mixed Model Fit by Maximum Likelihood (Laplace Approximation), Using Logistic Link in Experiment 2.***

***Formula: Acuraccy ~ Angle + (1 | Participant_number) (M2)***

|  | β | SE | *z* | *p* |
| --- | --- | --- | --- | --- |
| (Intercept) | 3.30 | 0.14 | 24.20 | < .001 |
| Angle90 | -0.11 | 0.09 | -1.27 | .20 |
| Angle135 | -0.30 | 0.09 | -3.40 | .001 |
| Angle180 | -0.22 | 0.09 | -2.45 | .01 |
| Angle225 | -0.49 | 0.08 | -5.82 | < .001 |
| Angle270 | -0.14 | 0.09 | -1.58 | .11 |
| Angle315 | 0.22 | 0.10 | 2.28 | .02 |

**3.** **Regression Analyses Concerning the Relationship between Accuracy and Speed**

**Supplementary Table S9**

***Regression Analyses Concerning the Relationship between Accuracy and Speed Using Logistic Link in Experiment 1a.***

***Formula: Accuracy ~ 1 + RT + (1 | Participant)***

*AIC = 18829.8*

|  | β | *SE* | *z* | *p* |
| --- | --- | --- | --- | --- |
| (Intercept) | 2.27 | 0.12 | 18.49 | < .001 |
| RT | 0.51 | 0.06 | 8.96 | < .001 |

*Correlation of Fixed Effects:*

*(Intr)*

*RT -0.37*

**Supplementary Table S10**

***Regression Analyses Concerning the Relationship between Accuracy and Speed Using Logistic Link in Experiment 1b.***

***Formula: Accuracy ~ 1 + RT + (1 | Participant)***

*AIC = 20741.5*

|  | β | *SE* | *z* | *p* |
| --- | --- | --- | --- | --- |
| (Intercept) | 1.76 | 0.09 | 17.84 | < .001 |
| RT | 0.89 | 0.05 | 18.56 | < .001 |

*Correlation of Fixed Effects:*

*(Intr)*

*RT -0.41*

**Supplementary Table S11**

***Regression Analyses Concerning the Relationship between Accuracy and Speed Using Logistic Link in Experiment 2.***

***Formula: Accuracy ~ 1 + RT + (1 | Participant)***

*AIC = 14369.8*

|  | β | *SE* | *z* | *p* |
| --- | --- | --- | --- | --- |
| (Intercept) | *2.94* | *0.14* | *21.53* | < .001 |
| RT | *0.23* | *0.07* | *3.55* | < .001 |

*Correlation of Fixed Effects:*

*(Intr)*

*RT -0.40*

**4.** **Results of the Questionnaire Data Analyses**

**Experiment 1a**

We found a significant positive correlation between the RTs and the scores of the perspective taking (PT) scale of the IRI, *τ* = 0.17, *p* = .04. The total score of the IRI, *τ* = 0.02, *p* = .76, and the scores of AQ, *τ* = -0.03, *p* = .69, did not correlate significantly with the RT data.

The questionnaires’ score correlations with the proportion of correct responses were not significant: PT scale in the IRI, *τ* = -0.09, *p* = .28; total score of the IRI: *τ* = -0.0007, *p* = .99; AQ score, *τ* = 0.07, *p* = .38.

**Experiment 2**

We found a significant positive correlation between the RTs and the total score of the IRI, *τ* = 0.24, *p* < .01. The PT subscale of the IRI, *τ* = 0.11, *p* = .21, and the scores of AQ, *τ* = -0.10, *p* = .21, did not correlate significantly with the RT data.

The questionnaires’ score correlations with the proportion of correct responses were not significant: PT scale in the IRI, *τ* = 0.02, *p* = .82; total score of the IRI: *τ* = -0.16, *p* = .05; AQ score, *τ* = - 0.05, *p* = .54.
